# Perception of Altered Smile Esthetics by Orthodontists, General Dentists, and Laypeople -

**DOI:** 10.31661/gmj.vi.3947

**Published:** 2025-11-08

**Authors:** Ozra Niknam, Shole Shahi, Jale Narimisaei, Mohabbat Mousaei Emami

**Affiliations:** ^1^ Department of Orthodontics, School of Dentistry, Ahvaz Jundishapur University of Medical Sciences, Ahvaz, Iran; ^2^ Department of Computer, Energy and Data Science Faculty, Behbahan Khatam-Alanbia University of Technology, Behbahan, Iran

**Keywords:** Smiling, Esthetics, Dental, Dentists, Orthodontists

## Abstract

**Background:**

Considering the significance of creation of a consonant smile arc and gap of information on the role of smile arc, gingival margin position, and the golden ratio in smile esthetics, this study assessed the perception of laypeople, general dentists, and orthodontists from altered smile esthetics.

**Materials and Methods:**

This descriptive study was conducted in 2019 with three rater groups: orthodontists (n=31), general dentists (n=49), and laypeople (n=61). A standardized frontal-view smile photograph of a female subject was digitally altered using Photoshop (version 19) to create images differing in (a) gingival margin position (four variations), (b) golden ratio (62%, 70%, and 80%), and (c) smile arc curvature (five variations). Raters, blinded to the alterations, evaluated each image’s attractiveness using a 10-point Likert scale. Due to non-normal data distribution, Kruskal–Wallis, Mann–Whitney, and Friedman tests were used for analysis (α=0.05).

**Results:**

The highest overall attractiveness ratings were given to the image with equal gingival margins for central and lateral incisors (M=7.16 ± 2.04), followed by the lateral margins 1 mm below the centrals (M=7.04 ± 2.01). Wider golden ratios (80%) were rated more attractive across all groups. Laypeople rated flat and reverse smile arcs significantly higher than general dentists and orthodontists (P.05). No significant gender-based differences were observed in any category.

**Conclusion:**

The three rater groups had the same opinion regarding the smile attractiveness of most altered images except for the reverse smile arc, which was only favored by the laypeople.

## Introduction

Facial attractiveness affects not only the self-esteem, but also the social interactions of individuals, and can influence their psychosocial well-being [1][2][3][4]. In fact, attractive individuals are generally better accepted by the community, have better communication with others, and are often more successful in education, job interviews, and even marriage [2][4][5]. Consequently, they are usually popular, sociable, and desirable [5].

A beautiful smile has a prominent role in facial attractiveness [1]. Indeed, the smile ranks next after the eyes in terms of having the greatest influence on facial beauty [6], and can even mask facial asymmetries to some extent [7]. In recent years, a growing attention has been directed to smile attractiveness, resulting in an increase in demand for orthodontic treatment [3][4][8]. Furthermore, with advances in dental science and a reduction in caries prevalence, the demand for cosmetic dental procedures is on the rise [9], and patients often appraise the treatment outcome according to the positive changes made in their smile [10].

The primary goal of orthodontic treatment is to achieve a functional and esthetically pleasant dentition and occlusion as well as an attractive smile [2]. Since anterior teeth are often the center of attention of observers, patients, and dental clinicians, their esthetic appearance is of utmost importance [9].

Given these considerations, dental clinicians should have sufficient knowledge about the principles of dental and facial esthetics to meet patient expectations [9]. However, it should be noted that perception of esthetics is a cognitive concept that may vary from one individual to another [3][11]. This perception is influenced by culture, ethnicity, social class, gender, personal experiences, and age [3][5][9][11]. As a result, some disagreements may exist in perception of esthetics between laypeople and dental clinicians [11]. In addition, dental education changes the criteria used for rating esthetics by dental clinicians and may be responsible for some differences in esthetic opinion between dentists and laypeople [4]. Therefore, what is believed to be ideal from the perspective of dental clinicians may be suboptimal according to the opinion of patients [1][12].

A number of dental and soft tissue parameters may affect smile esthetics [2]. Nevertheless, it is not known which parameter has the greatest role in this regard [7][12]. Broadly speaking, a beautiful smile depends on three key factors: the lips, gingiva, and teeth [1]. Smile analysis often includes evaluation of the smile arc, tooth show, gingival show, buccal corridor width, coincidence of facial and dental midline, dental ratios, tooth shade, gingival esthetics, and rotations of the occlusal plane [3][9][13]. Hence, dental clinicians should take into account all of the abovementioned factors in treatment planning [13]. A dental arch is optimal when the curve line passing through the incisal edge of the maxillary anterior teeth is parallel to the lower lip curvature [9]. Regarding the assessment of the position of the gingival margin, it should be noted that the central incisors often have the highest level of gingival margin. The gingival margin of the lateral incisors is often located 1.5 mm lower than that of the central incisors, and that of the canine teeth is often at the level of the central incisors [14].

To improve esthetics, the dimensions of the adjacent teeth in the dental arch should be proportionate. Ideally, the maxillary anterior teeth should follow the golden ratio. That is, the lateral incisor show from the frontal view should be 62% of the width of the central incisor, the canine tooth show should be 62% of the width of the lateral incisor, and the first premolar show should be 62% of the width of the canine tooth [2].

It appears that laypeople can also recognize the characteristics of an ideal smile. However, some dental clinicians do not correct small asymmetries since they believe that patients do not recognize them [9]. Given the importance of creating a consonant smile arc [14] and the existing lack of studies on the combined role of smile arc, gingival margin position of the anterior teeth, and the golden ratio in smile esthetics, this study aimed to assess the perception of laypeople, general dentists, and orthodontists regarding altered smile esthetics.

## Materials and Methods

This descriptive study was conducted on orthodontists, general dentists and laypeople in 2019.

### Sample Size

The sample size was calculated to be 36 in each group according to Oz et al. study [15] assuming alpha=0.05, study power of 90%, mean values of visual analog scale of attractiveness of 35.57 and 26.10 in the two groups and standard deviations of 7.15 and 19.84 in the two groups, per-calculations done in our previous study [16].

### Creating the Altered Images

**Figure-1:**
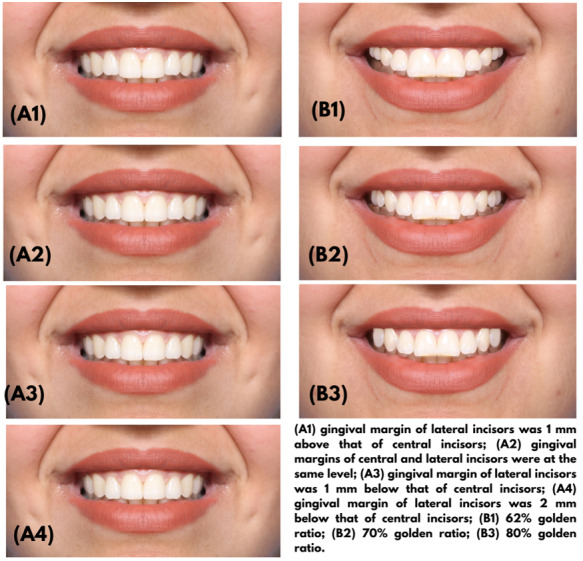
Figure 1. (A1) gingival margin of lateral incisors was 1 mm above that of central incisors; (A2) gingival margins of central and lateral incisors were at the same level; (A3) gingival margine of lateral incisors was 1 mm below that of central incisors; (A4) gingival margin of lateral incisors was 2 mm below that of central incisors; (B1) 62 % golden ratio; (B2) 70% golden ratio: (B3) 80% golden ratio.

A frontal-view photograph with a posed smile was obtained from an average-looking woman after signing written informed consent form. She had Angle’s Class I occlusion, no crowding, no spacing, a symmetrical normal smile, normal overbite and overjet, no dental implant or prosthetic restoration, normal 2-mm gingival show in a social smile, and normal facial size and ratios. The photograph was cropped from below the nose to below the chin to only visualize the smile. Next, Photoshop version 19 software was used to alter the following parameters on the original image:

Position of the gingival margin (Figure-1): The superior-inferior position of the gingival margin was altered in maxillary central and lateral incisors to create the following four images:

In the first image (A1), the gingival margin of the lateral incisors was positioned 1 mm above the gingival margin of the central incisors.

In the second image (A2), the gingival margins of the central and lateral incisors were at the same level.

In the third image (A3), the gingival margin of the lateral incisors was 1 mm below the gingival margin of the central incisors.

In the fourth image (A4), the gingival margin of the lateral incisors was 2 mm below the gingival margin of the central incisors.

**Figure-2:**
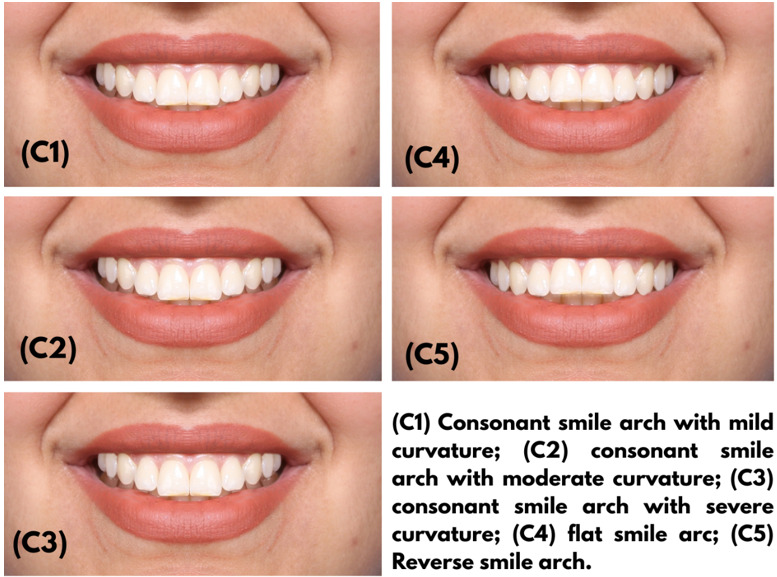
Figure 2. (C1) consonant smile arch with mild curvature; (C2) consonant smile arch with moderate curvature; (C3) consonant smile arch with severe curvature; (C4) flat smile arc; (C5) reverse smile arch.

Golden ratio (Figure-1): Three photographs were created such that the first image (B1) showed 62% golden ratio, the second image (B2) showed 70% golden ratio, and the third image (B3) showed 80% golden ratio. Smile arc (Figure-2): Five photographs were created with an altered smile arc. In the first (C1), second (C2), and third (C3) photographs, the incisal edges of the six maximally anterior teeth were parallel to the lower lip curvature, creating a consonant smile arc with mild, moderate, and severe curvature, respectively. The fourth photograph (C4) showed a flat smile arc such that the incisal edges of the maxillary anterior teeth formed a flat line and did not follow the lower lip curvature. The fifth image (C5) showed a reverse smile arc, such that the incisal edges of the maxillary anterior teeth had a reverse curvature relative to the lower lip curvature.

### Ratings

Three rater groups of orthodontists, general dentists, and laypeople (n=60 from each group) were selected for this study. Images were randomly arranged in a photo album in the form of an online questionnaire using Google Forms and sent to the raters. The raters were not aware of the altered parameters in each image. The raters were asked to rate the level of smile attractiveness of each image using a 1-10 likert scale such that 1 indicated the least attractive and 10 indicated the most attractive smile.

### Statistical Analysis

Due to non-normal data distribution as shown by the Kolmogorov-Smirnov test (P<0.05), general comparisons were made by the Kruskal-Wallis test, and pairwise comparisons were carried out by the Mann-Whitney test and Friedman test using SPSS version 22 (SPSS Inc., IL, USA) at 0.05 level of significance.

## Results

**Table:** Table1. Mean Attractiveness Scores (1-10) ± SD, Rank, and Significance

	**Laypeople (n=61) Mean ± SD (Rank) **	**General Dentists (n=49) Mean ± SD (Rank) **	**Orthodontists (n=31) Mean ± SD (Rank) **	**p**	**Female (n=88) Mean ± SD **	**Male (n=53) Mean ± SD **	**p**
			**Gingival Margin **				
**A1**	6.9 ± 2.4 (3)	7.2 ± 2.1 (1)	6.9 ± 1.7 (2)		7.1 ± 2.3	6.8 ± 1.8	0.289
**A2**	7.2 ± 2.3 (1)	7.2 ± 1.9 (1)	6.9 ± 1.5 (2)	>0.05	7.2 ± 2.1	7.0 ± 1.8	0.507
**A3**	7.0 ± 2.2 (2)	6.9 ± 2.0 (2)	7.1 ± 1.6 (1)		7.2 ± 2.0	6.6 ± 1.9	0.076
**A4**	6.5 ± 2.2 (4)	6.5 ± 1.9 (3)	6.5 ± 1.5 (3)		6.5 ± 2.1	6.5 ± 1.8	0.995
			**Golden Ratio**				
**B1 (60%) **	4.6 ± 2.4 (3)	3.6 ± 1.8 (3)	3.8 ± 2.0 (3)		4.1 ± 2.2	4.1 ± 2.1	0.795
**B2 (70%) **	5.4 ± 2.3 (2)	5.0 ± 1.9 (2)	5.3 ± 1.7 (2)	>0.05	5.1 ± 2.1	5.4 ± 2.0	0.414
**B3 (80%) **	6.1 ± 2.0 (1)	5.3 ± 1.9 (1)	5.5 ± 1.8 (1)		5.5 ± 2.1	6.0 ± 1.7	0.132
			**Smile Arc**				
**C1 (mild) **	6.1 ± 2.2 (4)	6.1 ± 1.7 (2)	6.2 ± 1.9 (1)	>0.05	6.0 ± 2.2	6.4 ± 1.5	0.35
**C2 (moderate) **	6.3 ± 2.3 (2)	5.8 ± 1.7 (4)	5.8 ± 1.7 (2)	>0.05	5.9 ± 2.2	6.3 ± 1.7	0.234
**C3 (severe) **	6.2 ± 2.2 (3)	6.0 ± 2.1 (3)	5.5 ± 1.9 (3)	>0.05	5.9 ± 2.2	6.2 ± 1.9	0.344
**C4 (flat) **	7.0 ± 2.0 (1)*	6.2 ± 2.2 (1)*	5.3 ± 1.7 (4)*	<.01	6.4 ± 2.2	6.3 ± 2.0	0.993
**C5 (reverse) **	7.0 ± 2.3 (1)*	5.8 ± 2.2 (4)*	5.2 ± 2.3 (5)*	<.01	6.1 ± 2.5	6.3 ± 2.1	0.624

Ranks are within each rater group (1 = highest attractiveness). ^*^ Indicates significant differences among rater groups at P<.05. Gender P-values are from Mann–Whitney tests.

A total of 141 participants completed the survey, including 61 laypeople (14 males, 47 females), 49 general dentists (23 males, 26 females), and 31 orthodontists (16 males, 15 females).

### Overall Ratings

Across all images, the highest mean attractiveness score was obtained for the image with equal gingival margin levels of central and lateral incisors (A2; M =7.16 ± 2.04), followed by the image with the gingival margin of lateral incisors 1 mm below the centrals (A3; M=7.04 ± 2.01). The lowest score was observed for the image where the lateral gingival margins were 2 mm below the centrals (A4; M=6.56 ± 1.99). Regarding the golden ratio, mean ratings progressively increased with higher ratios: 60% (B1; M=4.14 ± 2.21), 70% (B2; M=5.27 ± 2.09), and 80% (B3; M=5.76 ± 2.02). Among the smile arc variations, flat (C4; M=6.38 ± 2.12) and reverse (C5; M=6.21 ± 2.40) arcs received slightly higher ratings than the mild to severe consonant smile arcs (C1-C3; Ms=6.03-6.18 ± 2.00-2.13).

### Gingival Margin Position

When comparing gingival margin positions across the three rater groups, all groups consistently rated the image with the lateral incisors 2 mm below the central incisors (A4) as the least attractive. Laypeople (6.5 ± 2.2) and general dentists (6.5 ± 1.9) both gave their highest ratings to the image with equal gingival margins (A2; 7.2 ± 2.3 and 7.2 ± 1.9, respectively). Orthodontists, however, slightly preferred the image with the lateral gingival margins 1 mm below the centrals (A3; 7.1 ± 1.6). Despite these differences, the Kruskal-Wallis test revealed no statistically significant differences among the three rater groups for any of the gingival margin conditions, H(2)=1.73, P>.05, as shown in Table -1.

### Golden Ratio

For the golden ratio modifications, all groups showed a clear preference for wider ratios. Laypeople rated the 80% ratio (B3) as most attractive (6.1 ± 2.0), followed by 70% (B2; 5.4 ± 2.3) and 60% (B1; 4.6 ± 2.4). Similar trends were observed among general dentists (B3; 5.3 ± 1.9; B2; 5.0 ± 1.9; B1; 3.6 ± 1.8) and orthodontists (B3; 5.5 ± 1.8; B2; 5.3 ± 1.7; B1; 3.8 ± 2.0). The Kruskal-Wallis test showed no significant difference among the three professional groups in their assessment of golden ratio variations, H(2)=2.09, P>.05, as shown in Table-1.

### Smile Arc

For the smile arc modifications, laypeople gave the highest ratings to flat (C4; 7.0 ± 2.0) and reverse (C5; 7.0 ± 2.3) smile arcs, while general dentists favored the flat (6.2 ± 2.2) and mild curvature (C1; 6.1 ± 1.7) arcs. Orthodontists showed a clear preference for mild (C1; 6.2 ± 1.9) and moderate (C2; 5.8 ± 1.7) consonant smile arcs, and rated the reverse arc lowest (C5; 5.2 ± 2.3). The Kruskal-Wallis test indicated no significant difference among groups for consonant smile arcs (C1-C3; P>.05). However, significant group differences were found for the flat (C4) and reverse (C5) arcs, H(2)=6.84 and 8.12, respectively, both P<.05. Post hoc Mann-Whitney comparisons revealed that laypeople rated both the flat and reverse smile arcs significantly higher than general dentists and orthodontists (P<.05), while dentists also scored these images higher than orthodontists (P<.05), as shown in Table-1.

### Gender-based Differences

Comparison of ratings by gender revealed no statistically significant differences between male and female raters across any of the evaluated images (Ps>.05). For instance, mean scores for males and females were similar for gingival margin A2 (female: 7.2 ± 2.1; male: 7.0 ± 1.8; P=.507) and golden ratio B3 (female: 5.5 ± 2.1; male: 6.0 ± 1.7; P=.132). Similarly, no gender differences were detected in any smile arc image, including the flat (C4; P=.993) and reverse (C5; P=.624) configurations, as shown in Table-1.

## Discussion

This study assessed the perception of laypeople, general dentists, and orthodontists from altered smile esthetics. According to William et al. [14], 1 mm difference in the gingival margin level of central and lateral incisors is the most beautiful. However, the present results revealed that laypeople and general dentists gave the highest score to the image with the gingival margins of central and lateral incisors at the same level. Nonetheless, orthodontists preferred the image with the gingival margin of lateral incisor 1 mm below that of central incisor. All three rater groups gave the lowest score to the position of gingival margin of lateral incisor 2 mm below that of central incisor. Furthermore, in assessment of smile esthetics, general dentists and orthodontists were more sensitive than laypeople; however, the difference among the three groups was generally not significant. Sriphadungporn et al. [17] evaluated the opinion of raters from different age groups regarding the gingival show and gingival margin position, and found no significant difference between the young and old raters; although the younger group was more sensitive to the changes. Talic et al. [18] compared the opinion of laypeople and dentists regarding the gingival show and gingival margin position, and found no significant difference between them, which was in agreement with the present results. Cracel-Nogueira and Pinho [19] compared the opinion of laypeople, dental students, and general dentists regarding the parameters involved in smile esthetics such as the gingival margin position and dental diastema. They found that the three rater groups had different perceptions of smile attractiveness but with no significant difference. Also, age and gender of raters had no significant effect on their opinion. Their results were in accordance with the present findings. Mora et al. [1] compared the perception of dentists and laypeople regarding smile esthetics. They evaluated the effects of gingival margin position, midline shift, and gingival show on smile attractiveness. Dentists gave a higher score to smiles with the gingival margin of lateral incisors 1 mm shorter than that of central incisors while laypeople preferred the smile with the gingival margin of lateral incisors 1 mm higher than that of central incisors. In total, they found significant differences in the esthetic opinion of dentists and laypeople.

Comparison of smile attractiveness with 62%, 70%, and 80% golden ratios in the present study revealed that all three rater groups preferred 80% golden ratio followed by 70%. This result was in contrast to the findings of William et al. [14]. Orthodontists had the highest sensitivity in this regard. Saha et al. [20] compared the opinion of laypeople, general dentists, and dental specialists regarding some influential factors on smile attractiveness including the golden ratio and position of lateral incisors. They demonstrated that laypeople followed by general dentists were less sensitive and gave an acceptable score to a higher number of smiles while specialists were more sensitive and had stricter criteria, which was in agreement with the present findings.

According to William et al. [14], the most important parameter in determination of a beautiful smile is the smile arc. Orthodontists in the present study gave the lowest score to smiles with flat or reverse smile arc, and were more sensitive in this regard. They gave the highest score to smiles with mild and moderate smile arc. However, laypeople gave the highest score to flat and reverse smile arcs. General dentists believed that smiles with flat or mild smile arc were the most attractive while reverse smile arc was the least attractive. Almanea et al. [21] assessed the opinion of orthodontists, restorative dentists, and laypeople regarding the smile arc and reported that all three rater groups believed that a consonant smile arc was the most attractive; nonetheless, 27% of the laypeople in their study accepted the reverse smile arc as a beautiful smile. Parekh et al. [22] compared the opinion of laypeople and orthodontists regarding the smile arc and buccal corridor width. They showed that both rater groups gave the highest score to a consonant smile with minimal buccal corridor space, which was in contrast to the present findings, probably due to ethnic and racial differences between the two study populations.

The present study revealed no significant effect of gender of the raters on their opinion regarding parameters affecting the smile attractiveness, which was in agreement with the results of Ahrari et al, [2] in their study on the opinion of laypeople regarding the effects of buccal corridor and philtrum height on smile attractiveness.

This study had some limitations. Only a female’s smile was evaluated. Also, the opinion of only three rater groups was asked, and only the role of three parameters in smile attractiveness was analyzed. Also, a cropped image of smile was used, and age of the raters was not taken into account. Future studies are required on a male and a female’s smile, and are recommended to include the opinion of other specialists i.e., operative dentists and periodontists. Additionally, the role of other influential factors should be investigated, and full-face photographs may be used such that the raters could appraise the facial attractiveness as a whole. Furthermore, the effect of raters’ age on their opinion should be investigated.

## Conclusion

The three rater groups had the same opinion regarding the smile attractiveness of most altered images except for the reverse smile arc, which was only favored by the laypeople.

## Conflict of Interest

None.
